# Surface Protection Technology for Metallic Materials in Marine Environments

**DOI:** 10.3390/ma16206822

**Published:** 2023-10-23

**Authors:** Jing Xu, Hao Lu, Linxuan Cai, Yihong Liao, Jiadi Lian

**Affiliations:** 1 School of Mechanical Engineering, Hangzhou Dianzi University, Hangzhou 310018, China; xujing@hdu.edu.cn (J.X.); 222010125@hdu.edu.cn (H.L.); 212010072@hdu.edu.cn (L.C.); liaoyihong@hdu.edu.cn (Y.L.); 2 Department of Mechanical Engineering, China Jiliang University, Hangzhou 310018, China

**Keywords:** marine environment, abrasion, corrosion, erosion, surface technology

## Abstract

As the demand for the development and utilization of marine resources continues to strengthen, the service requirements for advanced marine equipment are rapidly increasing. Surface protection technology has become an important way of solving the tribological problems of extreme operating conditions and improving the safety performance of equipment by imparting certain special properties to the surface of the material through physical, chemical or mechanical processes to enhance the ability of the material to withstand external environmental factors. Combined with the extremely complex characteristics of the marine environment, this paper describes the commonly used surface protection technologies for metal materials in the marine environment. Research on surface texture was summarized under different surface reshaping technologies, as well as processes and coating materials under different surface modification technologies. Combined with the existing research progress and development trends of marine metallic materials, the surfaces of metal materials under the marine environment protection technology foreground are prospected and provide a reference for the improvement of equipment performance in extreme marine environments.

## 1. Introduction

With the depletion of land resources, countries are competing for marine resources [1]. All marine resource development and marine economic development activities cannot be separated from the support of marine engineering equipment such as marine vessels, submarines/submersibles, marine exploration and marine engineering. Marine exploration equipment under extreme conditions requires high reliability and a long service life. Tribological problems, especially abrasion, are one of the biggest challenges to improving the service performance of marine equipment. The research and development of marine equipment technology is limited by the development of marine materials. According to statistics [2], global natural disaster losses in 2018 reached USD 160 billion, but the cost of corrosion is much higher than this value, about USD 2.5 trillion. It is reported that the annual economic losses caused by friction and wear in developed countries in Europe and the United States account for about 2% to 7% of their GDP [3,4]. According to the China Marine Economy Statistical Bulletin 2021, the national marine GDP reached CNY 90385 billion in 2021 [5]. If frictional wear accounts for 4.5% of the GDP [6], the loss caused by the frictional wear of marine materials is about CNY 406.6 billion, of which marine corrosion loss accounts for about one-third of the total corrosion loss, reaching CNY 700 billion [7]. It is of great practical significance to improve the performance of marine materials and reduce the wear and tear of marine machinery and equipment to improve the economy of production.

The ocean is a multi-field coupled environment with high salt spray, high humidity, temperature alternation, load pressure, etc., which makes the dynamic service conditions such as friction, corrosion and microbial fouling of material surfaces/interfaces extremely complex. The wear and tear of engineering equipment working in seawater mainly comes from two aspects: one is the effect of solid particles in the fluid on the components, and the other is the load action of key friction sub-components. In marine environments, these two effects synergize with the corrosion of seawater to form corrosion–erosion interactions [8] and corrosion–load interactions [9]. The essence of failure is the reduction of contact material and deterioration of performance, which leads to the failure of marine equipment components. Modern surface technology needs to meet the requirements of marine engineering materials in harsh service conditions, extend the service life of the key components and improve the reliability and stability of the effective method [10], but it is also important to enhance the overall level of marine engineering equipment. However, there is a lack of overview of surface protection technologies, especially in the marine environment. This paper focuses on the surface protection technologies used to cope with marine abrasion problems in the marine environment. Analyzed the advantages and disadvantages of adopting different surface protection technologies. This paper is expected to supplement the lack of research review on surface protection technologies in the context of the marine environment, as well as to provide a reference for enhancing the service performance of equipment in an extreme marine environment.

## 2. Features of Extreme Marine Environment

**Figure 1:**
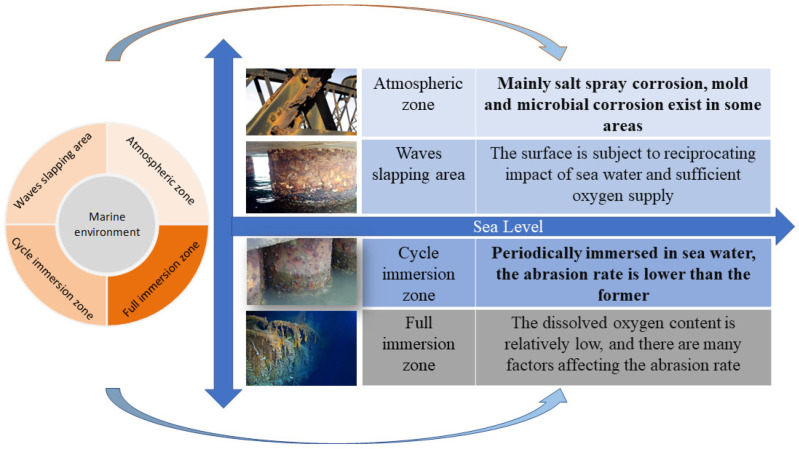
Abrasion characteristics of the marine environment.

The marine environment [11] includes seawater, substances dissolved and suspended in the seawater, seabed sediments and marine organisms. The extremely harsh environment includes wind, waves, currents, severe corrosion, high salinity, biological pollution, high temperature, high pressure, low temperature and high ultraviolet radiation. Such environmental factors have affected the material degradation and performance characteristics of offshore equipment. The ocean current speed, temperature, pH, salinity, dissolved oxygen content and microbial colonies at different sea areas and different depths are different, and the mechanisms of corrosion and erosion wear are also different. Only from the perspective of corrosion and wear is the ocean divided into four regions according to the ocean depth gradient, as shown in Figure 1. The corrosion effects, mechanisms and degrees in each region are different. They can be divided into four regions according to the ocean depth gradient: (1) Atmospheric zone [12]—the marine atmospheric zone contains various salt particles, and when metals are exposed to this environment, they absorb moisture from the atmosphere, resulting in strong corrosive effects on the metals. Furthermore, the microbial activity of fungi and mold that reside on the surface of the materials leads to the formation of a thin layer of electrolyte film on the metal surface. This provides favourable conditions for electrochemical corrosion. (2) Waves slapping area [13]—the steel surface is periodically wetted with sea water in this area [14]. The most serious corrosion occurs with the influencing factors of alternating states of dry and wet, sufficient oxygen supply, continuous concentration of salt [15,16], the synergistic effects of sunlight, wind and sea water environments and so on. In addition to the oxygen reduction reaction, the self-oxidation reaction of the rust layer is another main reason for accelerating the corrosion of steel structures in the spray splash zone. And the impact of a spray splash on the steel structure surface is also an important factor in intensifying corrosion damage. (3) Cycle immersion zone [17]—the metal components are immersed in sea water at high tide and exposed to air at low tide, with frequent alternations of dry and wet. Research shows that the corrosion degree of metal components in the ocean tidal range area is more serious than that in the atmospheric area and the full immersion area. (4) Full immersion zone [18,19,20]—the dissolved oxygen content is relatively low, and the corrosion rate is affected by temperature, pressure, salinity, marine organisms and bacteria. And the corrosion rate of materials increases with the increase in the dissolved oxygen, temperature and seawater velocity. In addition to biochemical corrosion, the vibration and erosion effects of wind, waves, currents, etc., on marine equipment cause accelerated wear of some connectors or weak friction pairs [21].

## 3. Current Situation of Surface Protection Technology

**Figure 2:**
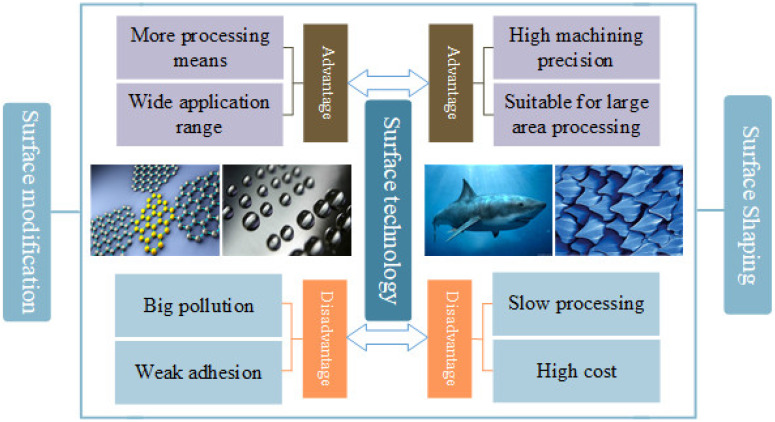
Advantages and disadvantages of surface technology.

The simple improvement of material properties and lubricating greases cannot quickly and efficiently meet the development of marine equipment. Surface technology focuses on the three factors of corrosion, wear and functional properties to change the chemical composition, organizational structure, surface state or form a special coating layer of the material surface via physical, chemical or mechanical processes so as to provide the material surface with some special properties and improve its ability to resist the action of the external environment. Surface technology refers to the engineering technology used to regulate and improve the performance of surface interfaces. It is mainly divided into surface modification technology and surface reshaping technology. Since the concept of surface engineering was first put forward in 1983, surface technology has developed into a comprehensive discipline spanning materials science, tribology, physics, chemistry, corrosion and protection, and it is widely used in the fields of anti-corrosion, wear resistance, repair and strengthening [22], which can effectively improve the safety and reliability of marine engineering equipment during service. The advantages and disadvantages of surface modification technology and surface reshaping technology are shown in Figure 2.

### 3.1. Surface Reshaping Technology

Surface reshaping technology mainly uses the surface micro-nano machining method to change the micro-geometry of the surface, that is, the application of surface texture, in order to reduce the abrasion loss of the equipment in the marine environment and improve its abrasion resistance.

#### 3.1.1. Research Progress of Surface Texture

Surface texture was first proposed by Dempster [23] in the mid-19th century, and it refers to the morphological deviation between the real surface and the reference surface. In 1997, Barthlott [24] first proposed that the self-cleaning effect of the lotus leaf was caused by its surface microstructure and waxy substances, and the surface micro-nanocomposite structure was proven to be the root cause [25]. Since 2001, people have been creating micro textures on the surface to improve the tribological properties of materials. Costa [26] concluded that texture has a significant effect on friction reduction under hydrodynamic lubrication.

Taking the actual friction pair of the equipment as an example, the surface texture can effectively reduce the surface wear of the friction pair and improve the bearing capacity of the lubricating oil film. Etsion [27] found that the surface texture produced via laser processing can reduce the friction of mechanical parts by up to 30–40%. The texture can increase the oil film gap, reduce the friction resistance and increase the lubrication effect [28]. Xu J. [29] processed micro-texture morphology on the surface of a marine directional valve spool, and the pressure adjustment time and vibration impact amplitude of the textured directional valve were reduced. Wang Jun [30] showed that the existence of optimal texture geometric parameters increases the bearing capacity of sliding bearings, reduces the friction coefficient and improves the performance of sliding bearings. Li Yunkai [31] added bionic pitcher structure to the water-lubricated bearing. The water-lubricated bearing modified by bionic-textured has better water film bearing capacity and antifriction performance than ordinary bearings.

Taking superhydrophobic surface drag reduction as an example, superhydrophobic surfaces are considered to be very promising for improving ship speed and navigation. Benschop [32] used embossing technology to manufacture textured coatings, and he found the resistance was reduced by up to 6%. Compared with parallel groove texture, herringbone texture will produce more secondary reflux. The use of convergent/divergent grooves in texture seems not conducive to drag reduction [33]. Xu J. [34,35,36] studied the morphological evolution and interface effect of droplet movement in transverse vibration of a micro-textured plate, results showed that the vortex pad effect and turbine effect are the main reasons for droplet acceleration; they reduce friction resistance and promote fluid movement.

Taking the surface anti-fouling and anti-corrosion of marine equipment as an example, biological fouling increases the ship’s own weight, hull surface roughness and hull corrosion, and greatly increases the ship’s fuel consumption and maintenance cost. Surface microstructure antifouling technology will not affect the environment, which is one of the research directions of ship green antifouling means at present. Fang [37] found that the removal rate of algal spores was significantly higher on terrain with complex surface structures. The biological adhesion of nanostructured surfaces is determined by structure and roughness. The surface structure has a high application prospect in decontamination.

#### 3.1.2. Surface Texture Processing Method

Surface texture has been successfully applied in many fields to improve surface properties, and many machining methods have been derived, mainly including laser machining technology [38], electrochemical machining technology [39], ultrasonic vibration machining technology [40] and so on [41,42,43].

Laser processing technology uses a laser beam to quickly and locally heat the workpiece to achieve the required surface morphology. Laser texturing has the advantages of fast processing speed, no pollution and stable control of micro-texture morphology. But it has the disadvantages of high cost, high requirements for workers and complex maintenance. Klink [44] used laser honing technology to process the micro-pit texture on the inner surface of the cylinder liner for the first time and conducted a comparative test, and the results showed that the micro-pit texture on the laser surface not only had a good anti-friction and anti-wear effect but also reduced the fuel consumption of the internal combustion engine by 20–25%. Sved [45] studies have shown that the ferrite phase in low carbon is transformed into a harder martensitic phase via direct laser ablation, and the high-strength grade phase of the material surface helps to improve the resistance. Wang [46] used a pulsed laser to process the circular texture on the end face of the seal ring. Under certain geometric parameters, the tribological performance of the textured seal ring is obviously better than that of the ordinary seal ring. Su [47] used laser to process micro grooves and micropores with different geometric characteristics on the surface of polycrystalline diamond. Results show that higher scanning speed, pulse frequency and lower average output power can reduce the size of micro texture and improve the morphology of micro texture at lower scanning speeds and higher average output power.

Electrical Discharge Machining technology refers to machining the workpiece in a certain medium through electric erosion. It can process the surface with complex shapes, and the tool is not in contact with the workpiece. It has the advantages of minimal force, short pulse discharge time, a good cooling effect and a small thermal impact on the processing surface. However, it is mainly used to process conductive materials such as metals, with slow processing speeds and certain electrode losses. Xiao [48] used EDM technology to prepare a micro-textured array on aluminum surfaces, which realized the superhydrophobic performance of aluminum surfaces and effectively improved the wetting and drag reduction characteristics of aluminum surfaces. Koyano [49] prepared micro-textured patterns of tens of microns on millimeter-level tool electrodes through special EDM.

Ultrasonic vibration machining technology uses ultrasonic frequency as a small-amplitude vibration tool to gradually break the surface through the hammering effect of an abrasive-free liquid. It is widely used for difficult machining materials, deep hole machining and thin-walled parts, but it is mainly suitable for machining brittle materials, For materials with small hardness and good plasticity, there is no obvious processing effect. Zhu Y. W. [50] used micro-ultrasonic composite EDM technology to process texture on metal surfaces and found that the surface quality of micro pits is good, and the pit depth is greater than that of single ultrasonic and ultrasonic EDM. Liu [51] proposed a new self-tuning ultrasonic elliptical vibration cutting technology to realize efficient Ductile Machining of microstructure arrays on brittle materials. The results show that this technology can improve machining efficiency by 30% while maintaining excellent surface roughness and crack-free surfaces.

Electrochemical machining technology is based on the principle of electrochemical corrosion in an electrolyte after anode metal is energized. It has great development potential in the field of micro manufacturing because it is not limited by the strength and hardness of the material itself, high productivity or good processing quality. However, it is difficult to ensure the stability in the processing process, and it is hard to recover and treat the electrolytic products. Mahata [52] processed an average diameter is 65 μm of the micro texture, and the replication of texture realized the graphics of micro pits and micro-square arrays.

**Figure 3:**
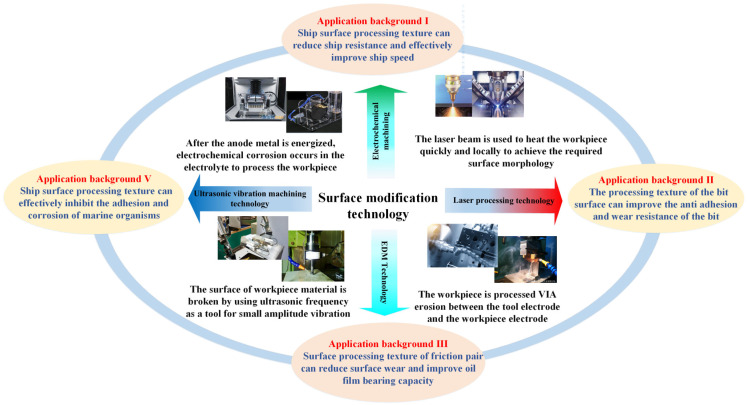
Processing method and application background of surface modification technology.

In view of this, surface modification technology effectively improves the service characteristics of marine equipment and is widely used, as shown in Figure 3. However, the complex marine extreme environment, such as the corrosivity of seawater and the changeable conditions such as wind, wave and marine fouling organisms, challenges the application of surface texture in the ocean. The problems of the long-term existence of surface texture, stable drag reduction and large-scale and effective processing seriously restrict the application of surface modification technology. How to process surface texture in a large range and with high efficiency and make it play its antifriction, antifouling and anti-corrosion performance in marine equipment stably and for a long time is an urgent problem to be solved in surface modification technology.

### 3.2. Surface Property Adjustment Technology

**Figure 4:**
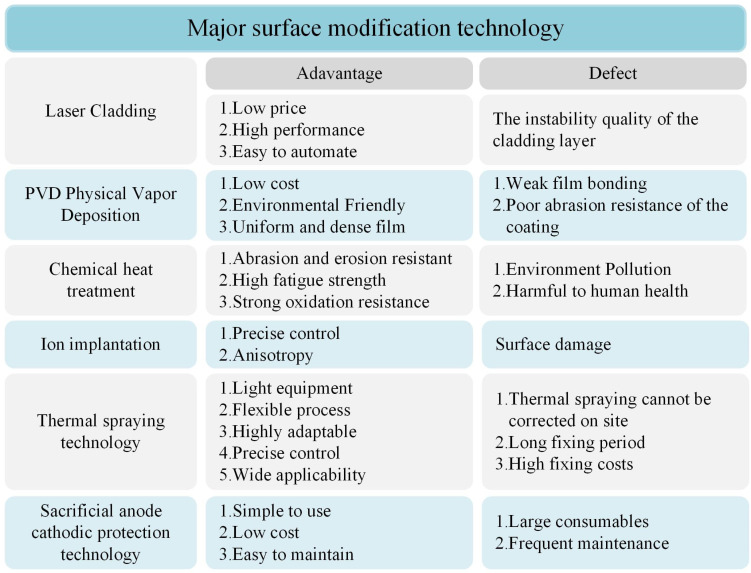
Application of surface property adjustment technology.

Surface property adjustment technology refers to changing the material and physical properties of the surface via coating or strengthening, such as corrosion resistance, wear resistance and photoelectric properties [53,54,55]. Adopting a reasonable surface treatment process can effectively improve the working stability and service life of the workpiece. Common surface modification technologies mainly include chemical heat treatment, thermal spraying technology, PVD, laser cladding and so on. The advantages and disadvantages of each surface property adjustment technology are shown in Figure 4.

#### 3.2.1. Chemical Heat Treatment Technology

Chemical heat treatment technology uses chemical reactions to change the chemical composition and microstructure of steel surfaces so as to obtain a better metal heat treatment process than homogeneous materials. The product processed in this way is a special composite material, which is much stronger than the combination of surface protection technologies such as electroplating. Tatsurom [56] carried out 43.2 k (12 h) plasma carburizing pretreatment at 773 k and then carried out diamond-like carbon (DLC) coating, and he showed that DLC coating has a remarkable effect on reducing friction coefficient, improving wear resistance and greatly improving fatigue strength (the improvement rate is 53%). Roa C. V. [57] studied the cavitation erosion and slurry jet erosion properties of steel surface coating and thermochemical treatment, and results show that ion nitriding can reduce the erosion rate of 13-4 steel by up to 96% under cavitation erosion while also reducing the rate of jet slurry erosion by 51%.

#### 3.2.2. Ion Implantation Technology

Ion implantation technology injects only a small number of elements without changing the matrix properties, obtains a highly supersaturated solid solution, a metastable phase and an amorphous structure [58] and effectively improves the wear, corrosion and oxidation resistance of the material surface [59]. Keshri A. K. [60] investigated the wear behaviour of plasma-sprayed carbon nanotube (CNT)-reinforced Al_2_O_3_ composite coatings in seawater and found that the addition of 8 wt% CNTs increased the wear resistance of Al_2_O_3_ (A-SD) coatings by 66% in seawater. This is because the coating coverage area increases, and the carbon nanotube bridging increases the fracture toughness of the AL_2_O_3_-CNT coating. Mateescu A.O. et al. [61] used cold plasma surface treatment to improve the mechanical, wear and protective properties of the surface of AISI304 stainless steel. The results showed that the surface friction coefficient of the treated material could be reduced to 0.2, the corrosion resistance was improved, and the surface hardness was higher than that of the untreated surface. Ye et al. have found that the nanocrystalline/amorphous structures and hard Cr7C3 phase formed by doping carbon elements in CrN coatings not only greatly improve the mechanical properties of the coatings but also enhance the coating’s friction and corrosion resistance in seawater due to the presence of the carbonaceous phase [62,63].

#### 3.2.3. Thermal Spraying Technology

Thermal spraying technology is a method of heating spraying materials to a molten or semi-molten state by using a heat source, then spraying and depositing them on the pretreated substrate surface at a certain speed to form a coating. This technology can manufacture functional surfaces with corrosion, wear, high temperature and oxidation resistance, heat insulation and insulation on the surface of ordinary materials. It can prepare various protective coatings and functional coatings on almost all solid material surfaces, with a wide range of applications. Huang J. [64] proved the Al-Al composite coating prepared using flame spraying technology has potential corrosion and wear resistance in the marine field. Meng Peiyuan [65] prepared the ultra-high molecular weight polyethylene/graphene composite coating on carbon steel substrate with flame spraying technology. When the mass fraction of graphene is 0.5%, the wear rate decreases by about 26%, which can effectively delay the corrosion and erosion of metal substrates with seawater and inhibit the serious damage to marine materials. Zulhelmia [66] showed the alumina coating can reduce the COF value and wear amount of the GCI substrate by at least 10% and 50%, respectively. Liyuan Xiao et al. [67] found that through the study of chemical changes in Al-Zn corrosion in the marine environment, Al-Zn coatings can generate a protective film of Al_2_O_3_ with good corrosion resistance during the corrosion process, forming a thick and dense layer of insoluble corrosion products, which reduces the diffusion of corrosive substances into the coating and inhibits the further occurrence of corrosion. Fu et al. [68] prepared Al-ND composite coatings via flame spraying, and the corrosion resistance and mechanical properties of the composite coatings were significantly improved due to the ND phase. It provides a promising method for nanomaterials to enhance the corrosion and wear resistance of metal-based composite coatings with potential marine applications. Zi heng Bai et al. [69] designed and synthesized a novel high-performance hydrophobic SC/SFPani@Zn coating to cope with harsh marine environments, which proved to be extremely corrosion-resistant in the harsh oxygen/artificial seawater environment. This excellent protection mechanism originated from the multifunctional linkage of the composite barrier properties, excellent shielding properties and unique corrosion inhibition properties, and this mechanism provided new ideas and strategies for the preparation of coatings with excellent corrosion protection properties.

#### 3.2.4. Physical Vapour Deposition (PVD) Coating Technology

Physical vapour deposition (PVD) coating technology can use physical methods (such as evapouration, sputtering, etc.) in a high vacuum environment to form coatings with specific surface requirements on the material surface at the atomic and molecular levels, so as to achieve high durability and low wear. Sun Hui [70,71] deposited TiCN and TAC coatings on stainless steel substrates via multi-arc ion plating and magnetron sputtering. They found the grain refinement caused by an appropriate amount of C and the disordered carbon at the grain boundary significantly affected the mechanical properties of the coating. Shan Lei [72] deposited CrN and CrAlN coatings on 316 L stainless steel via multi-arc ion plating, and results show that CrAlN coating has better wear resistance than CrN coating in a seawater environment. Li C. [73] deposited TiSiN, CrAlN/TiSiN bilayers and CrAlN/TiSiN multilayers on the surface of high-speed steel using multi-arc ion plating technology, and results show that the CrAlN/TiSiN multilayer coating improves the cohesion and bonding strength of the coating by reducing shear stress and has a denser microstructure than the other two coatings. Wang, Y.X. et al. [74] successfully prepared CrN, CrCN monolayer and CrN/CrCN multilayer coatings on the surface of 316 L stainless steel using multi-arc ion plating technology. The microstructure, mechanical properties and frictional corrosion behaviour of the coatings in artificial seawater were systematically investigated. The experimental results show that the corrosion resistance of CrN/CrCN multilayer coatings is much higher than that of CrN and CrCN monolayers and that CrN/CrCN multilayer coatings are an effective strategy to improve the frictional corrosion performance in the marine environment.

#### 3.2.5. Laser Cladding Technology

Laser cladding technology refers to a surface coating in which coating materials are placed on the coated surface in different filler ways, melted simultaneously with a thin layer on the substrate surface via laser irradiation and solidified rapidly so as to improve the wear, corrosion, heat and oxidation resistance of the substrate surface. It can prepare a cladding layer on a low-cost metal matrix, improve the performance of the component surface, carry out selective deposition, reduce material consumption and have an excellent performance price ratio, and the process is easy to automate. He X. [75] prepared a composite ceramic film on the surface of S355 marine steel by combining laser cladding and micro-arc oxidation technology. It is found that the composite film is well combined with the base layer, and its hardness is significantly higher than that of the cladding coating. When the current density is 5A/dm^2^, the composite film can significantly improve the wear resistance and corrosion resistance of the substrate and cladding coating. Wang S. W. [76] designed the laser-cladding Ni625/WC composite coating, which increased the surface hardness of cast iron matrix by 50% and reduced the wear amount to 5–10% of the matrix, improved the wear resistance and prolonged their service life. Kong D. J. and Chen H. X. [77] investigated the effect of laser power on the electrochemical corrosion behaviour of Al-Ti-Ni coatings in salt, acid and alkali solutions, where the best effect was achieved at a laser power of 1700 W. This study provides an experimental basis for the application of aluminium–titanium–nickel amorphous coatings in marine environments.

#### 3.2.6. Sacrificial Anode Cathodic Protection Technology

Sacrificial anode cathodic protection technology refers to the connection of the protected metal with a more active metal, the external metal, as the anode to withstand the corrosion reaction so as to achieve the protection of the cathode [78]. This method uses methods of simplicity, low cost, easy maintenance and the ability to increase the anode and marine corrosion prevention technology in a big way. Zakowski A. [79] introduced a cathodic protection system for struts in the Baltic Sea, using a conical sacrificial anode structure, and by comparing the results of measurements over a long period of time, it was concluded that the use of a sacrificial anode system mounted on the seabed is an effective form of cathodic protection for struts on offshore platforms. Erdogan C. [80] introduced a sacrificial anode cathodic protection system using a coating at the same time, and it was shown through experiments that the application of the coating system provided significant benefits in terms of the quality of the sacrificial anodes required to protect the structure. anode quality aspects, providing significant benefits in terms of reduced anode mass and cost and reduced aluminum emissions from the sacrificial anode. However, the drawbacks of this approach are also evident in the marine environment, where the loss of sacrificial anodes is significant, requiring frequent replacement of sacrificial anode materials and also increasing the maintenance costs of the cathodic protection system. At present, there is a great need to reduce the corrosion rate of sacrificial anodes and prolong the service life of sacrificial anodes by developing high-performance corrosion-resistant anode materials and high-quality corrosion-resistant anti-corrosion coatings [81].

#### 3.2.7. Other Processes

In addition, surface property adjustment technology also has mechanical surface treatment—shot peening, electroless plating, micro-arc oxidation and other process methods. Mechanical surface treatment—shot peening—can induce phase transformation and microstructure change, making the surface plastic deform and form a strengthening layer with a certain thickness, so as to improve the fatigue strength and service life of parts. Juan G. [82] proposed a mechanical surface treatment—shot peening. It was found that shot peening can transform retained austenite and improve the surface hardness of materials. These two effects are helpful in improving the erosion wear performance of WCI.

Electroless plating is a plating method in which metal ions in the plating solution are reduced to metal and deposited on the surface of parts with the help of a reducing agent without external current. The process is widely used because of its uniform coating, good decoration, environmental protection and ability to improve the corrosion resistance, electrical conductivity and lubrication of products. Y. Shajari [83] prepared the nanostructure of Ni-B coating on the surface of nibral alloy, a marine propeller material, and the surface hardness increased from 410 to 788 and 1365 Vickers hardness. The coating and heat treatment improved the wear resistance. Dimitra Kourtidou et al. [84] used a dual process that comprised electrodepositing and pack cementation to prepare Ni-Al coatings, which were experimentally shown to exhibit excellent resistance to oxidation and corrosion in artificial marine environments.

Through the combination of electrolyte and corresponding electrical parameters, micro-arc oxidation grows ceramic films dominated by matrix metal oxides on the surfaces of aluminum, magnesium, titanium and their alloys by relying on the instantaneous high temperature and high pressure generated via arc discharge. The process has the advantages of a simple process, high production efficiency and environmental protection. It can also effectively improve surface performance. Zhang Y. L. [85] studied the TC17 titanium alloy and its surface micro-arc oxidation, and results show the oxide film on the surface of the TC17 matrix breaks at the beginning of friction, which makes it difficult to play an effective protective role. However, MAO coating is composed of rutile phase and SiO_2_ phase with high hardness, which can play an excellent role in wear-resistant corrosion in the process of friction corrosion.

## 4. Summary

The development and breakthrough of marine engineering materials are the foundation and precursor for the realization of marine science and technology innovation and sustainable marine development. The study of tribological problems and failure mechanisms of marine engineering materials in extreme marine environments has become one of the key technologies to be developed in the field of marine engineering. This paper reviews surface modification technology and surface remodelling technology, which have been shown to cope with material abrasion problems in marine environments. Surface modification technology and surface reshaping technology have been analyzed through the current research on the principle of abrasion [86,87,88].

Surface modification technology can effectively reduce or prevent the occurrence of marine corrosion by changing the chemical properties of the material surface. Surface modification techniques are more suited to the needs of specific marine environments as they treat only the surface and do not alter the volume or structure of the material at a relatively low cost. However, as surface modification techniques only treat the surface of the material and have a limited effect on improving the internal structure, they may not be able to completely solve the corrosion problem of the material. Nonetheless, surface modification techniques are preferred to improve the performance of materials. Among them, anticorrosion coatings, as one of the most direct, effective and economical anti-corrosion technologies, are currently the most important protection measure for marine metallic materials. The author believes that the future of surface modification technology should pay more attention to the research and development of innovative materials, such as marine corrosion-resistant polymer coatings and corrosion-resistant alloy materials, in order to make the protection of marine corrosion surfaces more efficient.

Surface reshaping technology can change the chemical properties of the material surface, such as increasing the ability of anti-oxidation, acid and alkali resistance, etc. The overall performance is more outstanding compared to the surface modification technology, which can more effectively reduce or prevent the occurrence of marine corrosion. At the same time, because surface reshaping technology is applicable to almost all kinds of materials, it has a high degree of versatility and applicability. However, because surface modification technology can change the shape and structure of materials, it may introduce some defects, such as cracks and deformations, which require further processing and repair. Moreover, the long-term survival of the existing surface texture, stabilization and drag reduction, large-scale processing and high cost are also serious constraints to the application of surface reshaping technology. The author believes that surface reshaping technology in the future should focus on improving the efficiency and controllability of the technology to meet the higher requirements of marine corrosion surface protection needs. At the same time, the combination with other technologies, such as the combination of surface coating and intelligent monitoring technology, will further enhance the development prospects of surface reshaping technology.

Under the new situation in the new era, we should consider the multi-factor environmental characteristics; systematically put forward the design guidelines of materials, interfaces and structures through the methods of surface diversification, interface multi-scale and functional gradient; propose new protection mechanisms and develop high-performance wear-resistant and corrosion-resistant materials that break through the service limit. At the same time, how to make use of big data to carry out intelligent operations, maintenance and monitoring to further improve material life depends on further research, with a view to breaking down the barriers between traditional disciplines and solving common problems in abrasion science.

## Data Availability

Not applicable.
